# Generation of competent bone marrow-derived antigen presenting cells from the deer mouse (*Peromyscus maniculatus*)

**DOI:** 10.1186/1471-2172-5-23

**Published:** 2004-09-30

**Authors:** Bennett J Davenport, Derall G Willis, Joseph Prescott, Regina M Farrell, Teresa A Coons, Tony Schountz

**Affiliations:** 1 Department of Biological Sciences, Mesa State College, 1100 North Ave., Grand Junction, CO 81501, USA; 2 Saccomanno Research Institute, St. Mary's Hospital, 2530 N. 8^th ^Street, Wellington Bldg. 4, Ste. 100, Grand Junction, CO 81501, USA; 3 Infectious Disease and Inflammation Program, Department of Pathology, University of New Mexico School of Medicine, Albuquerque, NM 87131, USA

## Abstract

**Background:**

Human infections with Sin Nombre virus (SNV) and related New World hantaviruses often lead to hantavirus cardiopulmonary syndrome (HCPS), a sometimes fatal illness. Lungs of patients who die from HCPS exhibit cytokine-producing mononuclear infiltrates and pronounced pulmonary inflammation. Deer mice (*Peromyscus maniculatus*) are the principal natural hosts of SNV, in which the virus establishes life-long persistence without conspicuous pathology. Little is known about the mechanisms SNV employs to evade the immune response of deer mice, and experimental examination of this question has been difficult because of a lack of methodologies for examining such responses during infection. One such deficiency is our inability to characterize T cell responses because susceptible syngeneic deer mice are not available.

**Results:**

To solve this problem, we have developed an in vitro method of expanding and generating competent antigen presenting cells (APC) from deer mouse bone marrow using commercially-available house mouse (*Mus musculus*) granulocyte-macrophage colony stimulating factor. These cells are capable of processing and presenting soluble protein to antigen-specific autologous helper T cells in vitro. Inclusion of antigen-specific deer mouse antibody augments T cell stimulation, presumably through Fc receptor-mediated endocytosis.

**Conclusions:**

The use of these APC has allowed us to dramatically expand deer mouse helper T cells in culture and should permit extensive characterization of T cell epitopes. Considering the evolutionary divergence between deer mice and house mice, it is probable that this method will be useful to other investigators using unconventional models of rodent-borne diseases.

## Background

Hantaviruses (family *Bunyaviridae*) are rodent-borne and can cause hemorrhagic fever with renal syndrome (HFRS^4^) or hantavirus cardiopulmonary syndrome (HCPS) [1]. While HFRS is usually associated with Eurasian hantaviruses, HCPS is caused by any of several recently described New World hantaviruses [2-4]. In North America, the great majority of HCPS cases have occurred in the western United States and Canada and were caused by Sin Nombre virus (SNV).

Patients afflicted with HCPS exhibit pronounced pulmonary inflammation due to capillary leak syndrome, with the consequent hypotension often leading to rapid decline and death [5]. Virus is found in the lungs of infected humans, but without discernible cytopathology, and mononuclear infiltrates are observed that produce proinflammatory cytokines, including IL-2, IL-4, IFN-γ, TNF and lymphotoxin (LT) [6], suggesting that HCPS is an immunopathologic response to the virus. To date, more than 370 infections with hantavirus have been documented in the United States, with a 36% fatality rate.

Deer mice (*Peromyscus maniculatus*) are the principal reservoir host of SNV [4,7]. As is usual with some natural hosts, SNV infection of deer mice does not result in discernible pathology [8]. Infection parallels that of humans, with virus infecting capillary endothelial cells in many tissues, including the lungs, but without conspicuous cytopathology. However, in contrast to human HCPS, no pulmonary inflammation, capillary leakage, or mononuclear infiltrates are observed, and most, if not all, deer mice remain persistently infected for the remainder of their lives [9].

Deer mice are among the most common mammals in North America, found from the subarctic to central Mexico, except for the Atlantic seaboard and the southeast United States where other peromyscine species predominate [10,11]. Serosurveys of natural rodent populations suggest that hantavirus infections occur throughout the range of deer mice [7,12-15], which thus poses a potential threat to individuals who are in contact with these rodents. In addition, deer mice and other peromyscine rodents have been shown to harbor other human pathogens [16-28].

Very little is known about the mechanism by which the deer mouse immune system engages SNV because few reagents and methodologies have been developed and no susceptible inbred deer mice are available. The only useful immunological data that can be acquired is by use of serology; infected deer mice produce a neutralizing IgG response that is inadequate to clear the virus [8,9,29]. We previously cloned several deer mouse cytokine genes [30-32], but quantitative assays for the detection of the expression of these genes have not been developed. These limitations have made it difficult to determine what immunological events occur that impair an effective immune response without pathology. In some viral infections, persistence has been shown to occur because of impairment of helper and cytotoxic T cell responses, antigen presenting cell (APC) function, and development of APC from bone marrow progenitors [33-37]. Currently, none of these functions can be evaluated in deer mice.

Recent advances in hematopoietic stem cell research have identified an important role for granulocyte-macrophage colony stimulating factor (GM-CSF) in the expansion and maturation of bone marrow cells into competent APC [38-40]. We previously cloned a partial cDNA representing deer mouse GM-CSF and found that one of its receptor-binding domains is nearly identical to that of the common laboratory house mouse (*Mus musculus*) [30]. This led us to hypothesize that house mouse GM-CSF, which is commercially-available, might be useful in expanding and differentiating deer mouse bone marrow cells into competent APC. If so, then it should be possible to generate large pools of APC from individual deer mice that could be aliquotted and frozen for use in long-term T cell cultures, which would preclude the necessity for inbred deer mice. We present evidence that such cells can be propagated in vitro and that they are capable of processing antigen and stimulating antigen-sensitized autologous T cells. This technique could provide sufficient APC, such that conventional T cell cloning and peptide-mapping experiments could be performed. In addition, because deer mice (New World rodents) and house mice (Old World rodents) are divergent by 25 to 50 million years [41], it is possible that this approach may be useful to investigators using other unconventional rodent models of infectious diseases.

## Results

### Cloning of the 5' end of deer mouse GM-CSF

**Figure 1:**
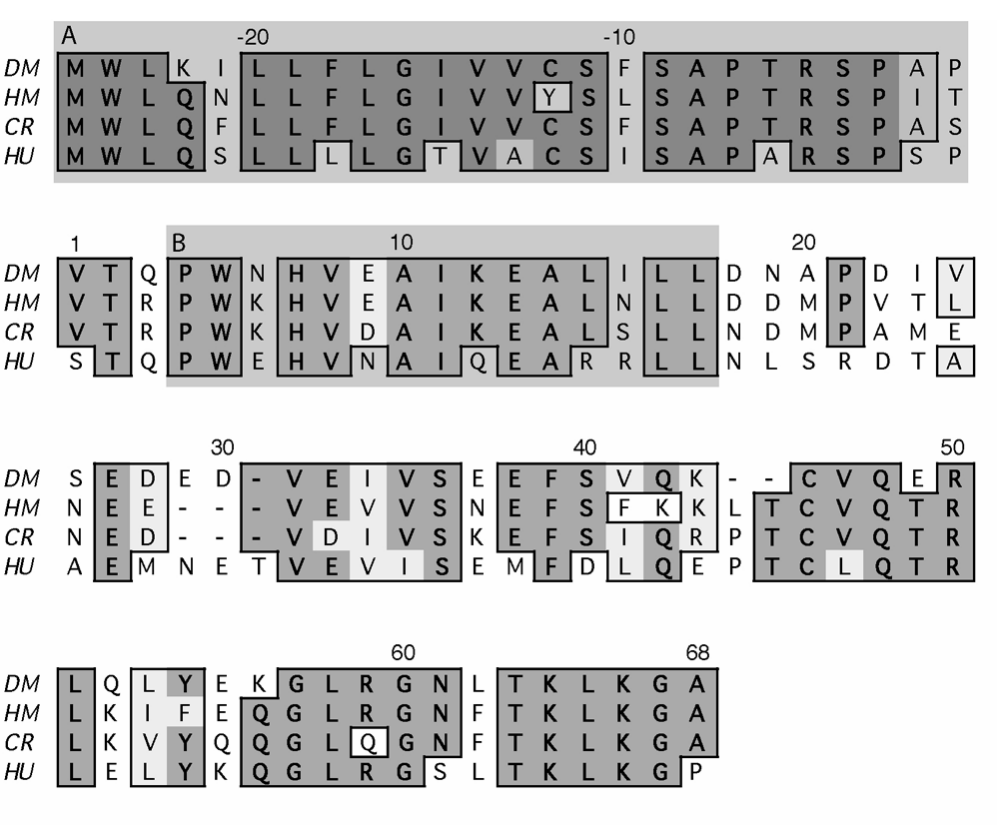
Amino acid alignment of deer mouse (DM), cotton rat (CR), house mouse (HM) and human (HU) GM-CSF. Polypeptides were aligned with the clustal algorithm in Macvector. Conserved (light shading) and identical (dark shading) amino acids are enclosed in boxes. The 25-residue signal peptide is enclosed in box A. Helix A, which binds to the β chain subunit of the GM-CSF receptor, is enclosed in box B. Deer mouse and house mouse GM-CSF share 13 of 15 identical residues in this domain.

We used RACE to obtain the complete 5' end of GM-CSF. This sequence was translated using the default translation table within MacVector. The polypeptide is predicted to have a 25 residue signal peptide based upon orthologous sequences from other species [42-45] (Figure 1). The receptor-binding domains of deer mouse and house mouse GM-CSF share 13/15 identical residues. This region forms the α-helix (helix A) that binds with high affinity to β chain subunit of the GM-CSF receptor complex that is shared with the IL-3 and IL-5 receptors [46-49].

### Morphologic characteristics of bone marrow-derived APC

**Figure 2:**
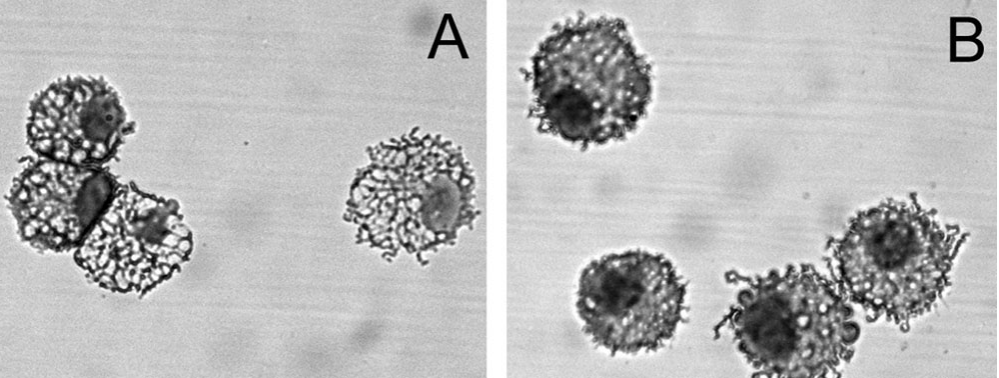
Morphologic characteristics of deer mouse bone marrow-derived APC. Day 14 bone marrow cells cultured in GM-CSF were processed by cytospin and stained with Wright's stain. The cells exhibited conspicuous cytoplasmic vesicles and small processes (A). Cells collected on day 12 and incubated for 48 hours with 20 ng/ml of hmTNF displayed less conspicuous cytoplasmic vesicles (B).

Deer mouse bone marrow cultures contained mostly cells that appeared dead or dying after 24 hours in culture with GM-CSF. However, at 48 hours clusters of cells were apparent, while control wells without GM-CSF had fewer live cells than at 24 hours. By day 3, adherent stromal cell foci were conspicuous, while semiadherent and nonadherent cells were more evident and these became the prominent cells for the duration of culture. Day 12 bone marrow cells incubated for an additional 48 hours were large, ranging from 12 to 18 μm in diameter, and possessed macropinocytic vesicles and processes (Figure 2A). Although the method that was employed selects for DC in the house mouse [39], the deer mouse cells appeared to resemble macrophages, with abundant cytoplasmic vesicles, rather than dendritic cells, with lamellipodia or characteristic long processes extending from the cell. (S. K. Chapes, pers. comm.). However, the cells' exact definition will not be complete until better phenotypic characterization is possible. Treatment of the cells with recombinant TNF diminished the macropinocytic vesicles (Figure 2B).

### Proliferation of deer mouse cells to house mouse GM-CSF and human IL-2

**Figure 3:**
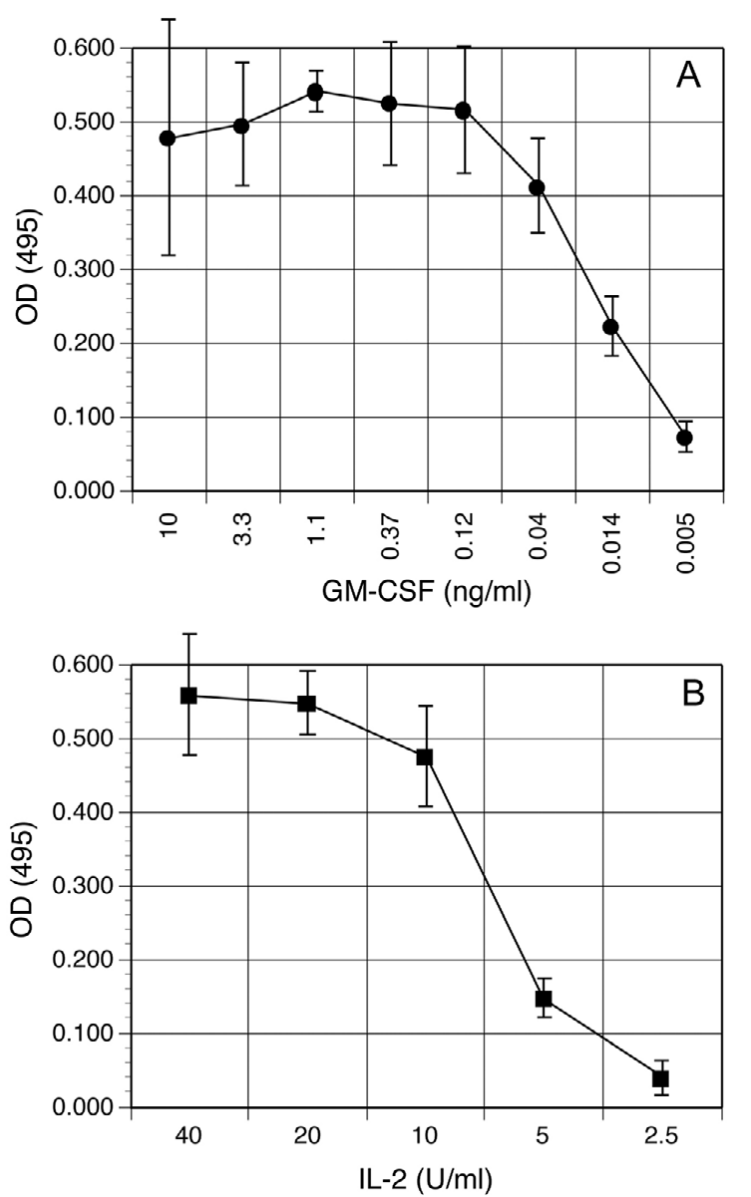
Proliferation of deer mouse cells to recombinant cytokines. (A) After 8 days of incubation with GM-CSF, deer mouse bone marrow cells were washed and then cultured with dilutions of GM-CSF in duplicate for 48 hours, then proliferation assessed by MTS assay. The data are representative of four deer mice. (B) To assess proliferative capacity of deer mouse T cells to human IL-2, splenocytes were cultured with a suboptimal dose of PHA (2 μg/ml) and dilutions of recombinant human IL-2 in duplicate for 48 hours, and proliferation assessed by MTS assay. The data are representative of two deer mice.

Day 8 deer mouse bone marrow cells were cultured with various concentrations of house mouse GM-CSF. Two days later, proliferation was assessed (Figure 3A) and maximal proliferation was observed at about 0.5 ng/ml of GM-CSF. Deer mouse splenocytes proliferated in response to human IL-2 (Figure 3B). In this experiment, deer mouse splenocytes were cultured with a suboptimal concentration of PHA and various concentrations of recombinant human IL-2. Maximal proliferation occurred at 20 U/ml of IL-2. In another proliferation assay, in vitro deer mouse T cells that were collected 8 days after stimulation with APC and antigen exhibited slightly greater proliferation to IL-2 (data not shown).

### Expression of MHC class II I-Eβ and TCRβC by deer mouse cells propagated in vitro

**Figure 4:**
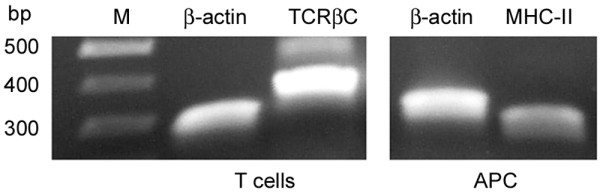
RT-PCR of TCRβC and MHC class II I-Eβ in deer mouse cells. Total RNA was extracted from T cells and bone marrow-derived APC. Expression of the constant β chain of the TCR by the T cells and class II I-Eβ chain by the APC were detected by RT-PCR. β-Actin primers were used as controls for each sample.

BM-APC and T cells were examined for the expression of orthologous I-Eβ and TCRβC, respectively, by RT-PCR (Figure 4). For I-Eβ, primers were designed from previously published deer mouse sequences [50], while primers for TCRβC were those that are described in this work. In each instance, products of the expected sizes were amplified. The amplified BM-APC product was cloned, sequenced, and verified to be I-Eβ.

### BM-APC induce antigen-specific proliferation of autologous T cells

**Figure 5:**
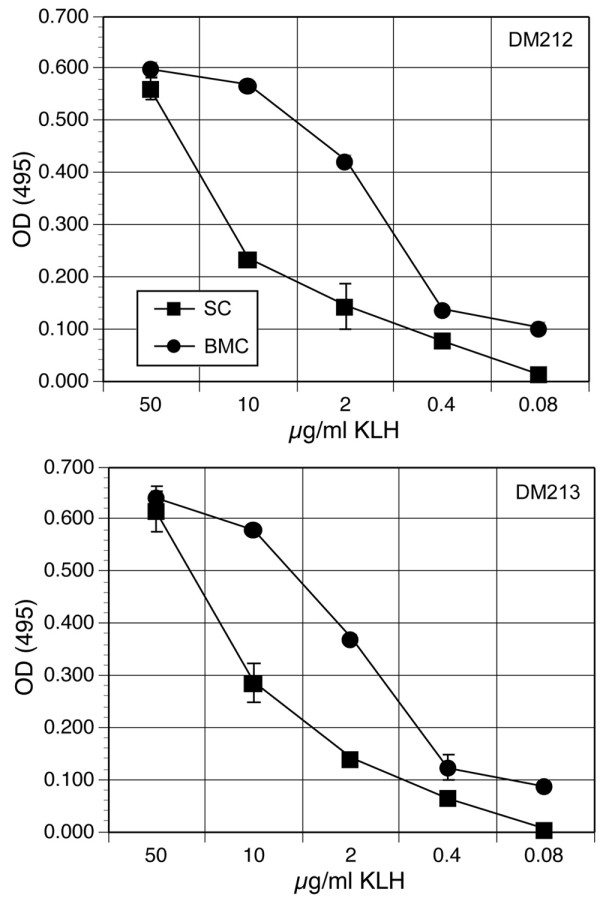
BM-APC stimulate helper T cell proliferation. Deer mice were immunized with 20 μg of KLH subcutaneously and 10 days later the lymph nodes, bone marrow and splenocytes were retrieved for expansion of helper T cells and BM-APC. After expansion of these cells in culture, proliferation assays were performed comparing mitomycin-C-treated autologous splenocytes (SC) and BM-APC (BMC) for their capacity to stimulate T cells. In each instance, the BM-APC were more effective at stimulating T cell responses. The data are of two deer mice (DM212 and DM213).

Deer mice were immunized with keyhole limpet hemacyanin (KLH), and 10 days later the lymph nodes, spleens and bone marrow were processed for in vitro expansion of polyclonal T cells (lymph node cells), while the bone marrow cells and splenocytes were frozen. Sera were tested for antibodies to KLH by ELISA and in each deer mouse tested the titer was greater than or equal to 8,000 (data not shown). For recall proliferation, KLH, in vitro-propagated T cells (14 days with IL-2) and BM-APC (14 days with GM-CSF) or freshly thawed splenocytes were cultured together for 72 hours, and proliferation was assessed by MTS assay (Figure 5). For each deer mouse, the BM-APC were between 10× to 20× more efficient at stimulating antigen-specific helper T cell proliferative responses. While each T cell line exhibited a 50% maximal stimulation in the presence of about 10 to 20 μg/ml antigen with splenocytes, 50% maximal stimulation was usually near 1 to 2 μg/ml antigen with BM-APC. In parallel experiments, cultures incubated with 20 ng/ml house mouse TNF did not exhibit noticeably different proliferative responses compared to control cultures (data not shown), despite morphological evidence suggesting an effect on macropinocytosis (Figure 2).

### Antibody augments BM-APC stimulation of T cells

**Figure 6:**
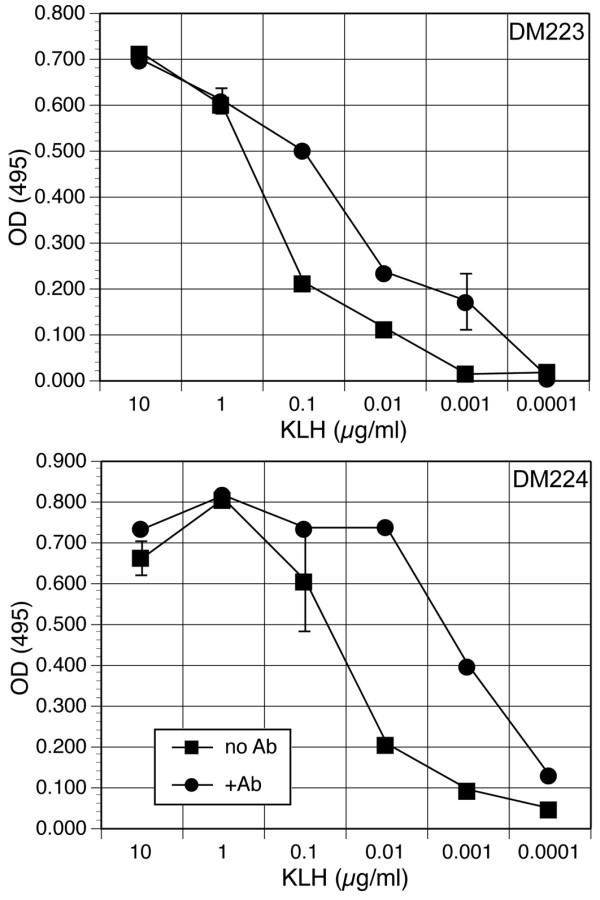
Antigen-specific antibody augments BM-APC-induced T cell proliferation. T cell proliferation responses from deer mice 223 and 224 were assessed as described in Figure 5 using BM-APC. KLH-specific antiserum or normal deer mouse serum were diluted 1:2,000 in DMM-5 and incubated with dilutions of KLH for 1 hour in 96-well plates at room temperature. BM-APC and T cells were added to the wells and incubated 72 hours, and proliferation was assessed by MTS assay.

Deer mouse antiserum raised against KLH and incubated with antigen for one hour prior to addition of cells increased the sensitivity of T cell proliferation (Figure 6), suggesting that Fc receptors are present on the surface of the BM-APC. The presence of antibody increased the 50% maximal T cell proliferation from one deer mouse (DM223) about 10-fold, from about 500 ng/ml without antibody to 50 ng/ml with antibody. Similarly, for another deer mouse (DM224) the presence of antibody was substantially more effective at inducing T cell proliferation, increasing 50% max 50-fold (from 50 ng/ml to about 1 ng/ml). Notably, the T cell response in DM223 was also less vigorous (50% max 500 ng/ml) without antibody compared to DM224 (50% max 50 ng/ml). The proliferative responses of six other deer mouse T cell lines were similar to those of these deer mice (data not shown).

## Discussion

To our knowledge, no previous efforts have been made to develop long-term cultures of T cells from unconventional laboratory rodents. The principal reason for this is that highly inbred strains, required for conventional long-term T cell work, are not available from rodents not routinely used in laboratory work. At least for deer mice, we have developed a method of fulfilling this need by using commercially-available house mouse GM-CSF. This cytokine apparently binds to the GM-CSF receptor on deer mouse cells such that it generates competent APC from the bone marrow. These cells are capable of processing and presenting soluble antigen to autologous antigen-specific helper T cells.

Our initial suspicions that house mouse GM-CSF might bind to deer mouse GM-CSF receptor was the result of previous work [30] in which we cloned a partial cDNA of deer mouse GM-CSF, including most of its A helix that is involved in binding to the β chain subunit of the receptor. We used 5' RACE to obtain the complete N-terminus and found that all but two of the residues from helix A are identical between the two species. Subsequent experiments demonstrated that GM-CSF induces proliferation of deer mouse bone marrow cells, and since GM-CSF is routinely used to generate APC from the bone marrow we hypothesized that it would do so with deer mouse bone marrow.

We used a method that has been shown to generate dendritic cells in house mice; however, the cells obtained from deer mouse bone marrow more closely resembled macrophages rather than DC. These cells contained many large macropinocytic vesicles, but conspicuous dendrites typical of DC were not observed. Microscopically, these cells also appeared sensitive to TNF, which decreased macropinocytosis, but it had no effect on the capacity of these cells to present antigen to T cells as has been reported for human DC derived from blood mononuclear cells [51]. TNF can induce a physiologic change in the APC from an active pinocytotic cell into one that becomes highly efficient at MHC class II antigen presentation, thus facilitating transition from the innate phase to the adaptive phase of the immune response. It is unclear why TNF treatment does not augment T cell proliferative responses with deer mouse APC, but it may be that species-specific differences in GM-CSF and TNF signaling occur that account for these disparities in APC development and behavior. It is also possible that TNF does not induce complete maturation of the cells into highly efficient APC, as has been reported for some DC [52]. It is currently impossible to phenotype these cells because no antibodies specific to deer mouse APC subpopulations, such as CD markers, are available, nor have genes for these markers been cloned, despite many attempts (unpublished observations), that might facilitate identification of these cells. Regardless, the cells are highly efficient at processing and presenting antigen, and inclusion of antigen-specific antibodies augments these functions.

**Figure 7:**
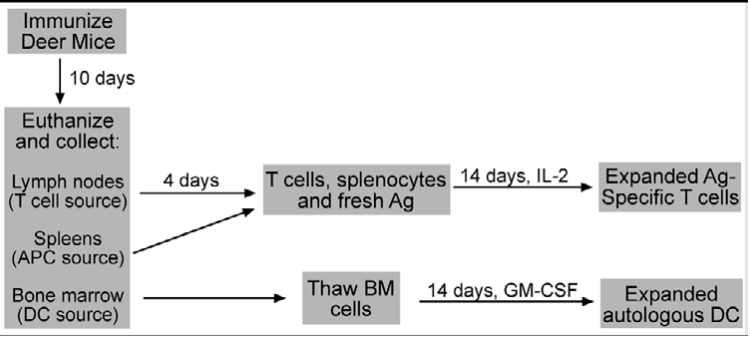
Schematic overview of the process for culturing autologous deer mouse BM-APC and T cells. Ten days post immunization, lymph nodes, spleens and bone marrow are harvested from euthanized deer mice. The lymph node cells are cultured with antigen for four days, while the splenocytes and bone marrow cells are frozen at -70°C. On day 4, the blasting lymph node T cells are recovered and cultured with fresh antigen and thawed splenocytes (without mitomycin-C treatment). Simultaneously, bone marrow cells are thawed and cultured with GM-CSF. Expansion of the T cells is performed with huIL-2 and the BM-APC with GM-CSF for 14 days. These cells are then used for proliferation experiments.

Since the deer mice are outbred, this method requires the immunization and collection of cells from individual animals (Figure 7). These cells are derived from lymph nodes (T cell source), splenocytes (APC source) and bone marrow (APC source). Most of the recovered cells can be propagated in vitro and/or aliquotted and stored frozen so that viable cells can be used as necessary to propagate and characterize helper T cell lines. We routinely recover 10^7 ^bone marrow cells from a deer mouse, which is sufficient for freezing 5 vials at 2 × 10^6 ^cells each. Each vial is used to seed a 100 mm bacterial Petri dish, which produces about 10^7 ^BM-APC at 14 days of culture. For deer mice, the most significant limitation for cells is from the spleen. Although deer mice are slightly smaller than BALB/c mice, their spleens are disproportionately small (unpublished observations). We routinely recover 7 × 10^6 ^splenocytes from a deer mouse, while BALB/c house mice usually provide 10-fold more. Because of this limitation, we have begun to use BM-APC to propagate T cells. This method involves culturing of bone marrow cells with GM-CSF for 10 days, then freezing aliquots of 10^6 ^cells. Three days before T cell restimulation, the 10-day BM-APC are thawed and cultured with GM-CSF, then used for restimulation with fresh antigen in one well of a 24-well plate. The T cells are fed fresh IL-2 DMM-5 at two-day intervals for expansion.

We have used this method to establish nine T cell lines, six specific for KLH and three specific for SNV nucleocapsid antigen (data not shown). Based upon typical cell yields, it should be possible to assay several thousand wells on 96-well plates, which we estimate to be sufficient for many T cell activities, including cloning, peptide epitope mapping, TCR variable gene segment usage, and cytokine profiling.

We believe the methods described in this work will allow the characterization of antigen presentation and T cell responses in infected deer mice. Many viruses impair pathways involved in APC and T cell functions so that they can evade a sterilizing immune response. With hantaviruses and their rodent hosts, millions of years of evolution have presumably allowed a coadaptation of the viruses and host immune responses such that pathology does not occur and the virus is not eliminated. It is possible that hantaviruses possess some as yet unidentified mechanism for suppressing an aggressive inflammatory immune response in rodent hosts, which is ineffective in human infections and often leads to inflammatory immunopathology.

Because of the substantial evolutionary divergence of deer mice and house mice (about 25–50 million years) [41], it is likely that this method could be used with many divergent rodent species, and thus useful for examining APC and T cell responses in a variety of rodent systems, including natural hosts and animal models of disease.

A limitation of this approach is that BM-APC lose their ability to proliferate in the presence of GM-CSF after four to six weeks, similar to what has been observed with house mouse bone marrow-derived cells [53,54]. Since a finite number of bone marrow cells can be harvested, this limitation prevents indefinite propagation of deer mouse T cells. It is possible that competent BM-APC might be propagated indefinitely by the introduction of oncogenes, such as transforming retroviruses [55]. Alternatively, other house mouse or human hematopoietic cytokines that are commonly used to propagate bone marrow progenitor cells may bind to deer mouse receptors. For example, human or house mouse Flt3 ligand is active on both human and house mouse cells, suggesting that one or both would have an effect on deer mouse cells as well. In this manner, large numbers of progenitor cells might be produced in vitro for storage and then thawed, as needed, for culturing in GM-CSF to produce functional APC.

## Conclusions

We have developed a method for generating large numbers of competent antigen presenting cells from deer mouse bone marrow using house mouse GM-CSF. This method resulted in the production of antigen-specific T cell lines from outbred deer mice. Inclusion of antigen-specific antibody in cultures augments T cell proliferation, suggesting the APC express Fc receptors. This method will allow characterization of APC and T cells in deer mice and may be extended to other rodent species that are important in infectious disease research.

## Methods

### Deer mice

The deer mice used in these experiments were from a colony of animals established with deer mice trapped in western Colorado [30]. All procedures were approved by the Mesa State College Institutional Animal Care and Use Committee and in accordance with the Animal Welfare Act.

### Cloning of the deer mouse GM-CSF 5' cDNA

The 5' end of the deer mouse GM-CSF cDNA was obtained by using RACE. Briefly, a primer (Table 1) was designed from a partial cDNA of deer mouse GM-CSF [30] and used to amplify the 5' end according to manufacturer's directions (SMART RACE, BD CLONTech, Palo Alto, CA) using con A-activated spleen cell culture cDNA. The fragment was cloned into pGEM-T Easy (Promega, Madison, WI) and sequenced using the Big Dye Terminator sequencing kit (Applied Biosystems, Foster City, CA) and an ABI 310 DNA Analyzer, and the sequence was deposited into Genbank (AY247762). The signal peptide and receptor-binding domain was identified by comparison to house mouse GM-CSF [49]. GM-CSF polypeptides from the house mouse (X02333), cotton rat (*Sigmodon hispidus*, AAL55394) and human (NP_000749) were aligned using MacVector's (Accelrys, San Diego, CA) clustal algorithm.

**Table 1:** Primers used in this work^1^

Gene	Forward	Reverse	Size (bp)
I-Eβ	GTC ATT TCT ACA ACG GGA CG	TCT CCG CTG CAC AAT GAA GC	242
TCRβC	AGG ACC TGA GCA AGG TGA GC	GCA CAG CAT ACA GGG TGG CC	474
β-Actin	ATG TAC GTA GCC ATC CAG GC	TCT TGC TCG AAG TCT AGG GC	283
GM-CSF 5' RACE	N/A	GTT GCC CCG TAG GCC CTT CTC ATA TAA CT	273

^1^All sequences are listed 5' to 3'.

### Cloning of the deer mouse T cell receptor β constant domain cDNA

Total RNA from activated splenocytes was reverse-transcribed using an oligo-dT primer and Superscript II (Invitrogen, Carlsbad, CA). TCRβC cDNA sequences from house mouse, rat and human were aligned with MacVector. PCR primers (Table 1) were designed from highly conserved regions within the alignment. PCR was performed on activated splenocytes with 95°C for 30 sec, 58°C for 30 sec, and 72°C for 1 min for 35 cycles. The amplified fragment was cloned and sequenced as described above, and deposited into Genbank (AY307417).

### Immunization of deer mice

Deer mice were bilaterally immunized subcutaneously at the base of the tail with 20 μg of KLH (Sigma Chemical Co, St. Louis, MO) emulsified in CFA (Sigma). Ten days later, draining lymph nodes, spleens and bone marrow were recovered for in vitro experiments. For production of high-titer KLH antiserum, deer mice were immunized i.p. with 20 μg of KLH emulsified in CFA and boosted with 20 μg in IFA one month later. Sera were collected 7 days after boosting.

### Processing of tissues from immunized deer mice

Immunized deer mice were euthanized by cervical dislocation and the draining lymph nodes, spleens and bone marrow from individual animals were separately collected in Hank's balanced salt solution for processing. The lymph nodes served as a source of antigen-specific T cells, while the splenocytes were treated with ammonium chloride (Cambrex Bioproducts, Walkersville, MD) to lyse RBCs, then frozen in 10% DMSO/5% FBS deer mouse medium (DMM-5: 5% FBS, RPMI-1640 supplemented to 315 mOsm [with 2.5 ml of 5 M NaCl/L], 2.5 μg/ml Fungizone, 100 U/ml penicillin, 100 μg/ml streptomycin, 50 μg/ml gentamicin, 50 μM β-ME, 10 mM HEPES, 2 mM L-glutamine) in aliquots for use as autologous APC for additional rounds of in vitro T cell stimulation. The bone marrow cells were collected from tibiae and femurs, washed twice in DMM-5, and aliquotted at 2 × 10^6 ^cells per vial in 1 ml of 10% DMSO/DMM-5 and stored at -70°C.

### Bone marrow culture

Bone marrow-derived APC (BM-APC) were generated with modification of a previously described method for dendritic cells (DC) [39]. One vial of bone marrow cells (2 × 10^6^) was quick-thawed in a 37°C water bath and cultured without washing in 100 mm bacterial Petri dishes in DMM-10 containing 20 ng/ml recombinant house mouse GM-CSF (R&D Systems, Minneapolis, MN) at 37°C under 7% CO_2_. Fresh GM-CSF/DMM-10 was provided on days 3, 6, 8, 10, and 12 for the generation of APC. Cells were collected with a scraper for use in experiments. Cells were processed by cytospin and stained with Wright's stain for morphological examination.

### Assessment of T cell sensitivity to human IL-2

Deer mouse splenocytes depleted of RBC by ammonium chloride treatment were incubated with a suboptimal dose of PHA (2 μg/ml) (Sigma) in DMM-5 and recombinant human IL-2 (R&D Systems) for 48 hours. Proliferation was determined by MTS assay (Cell Titer-96 AQ, Promega). The means and standard deviations of duplicate samples were calculated, with the mean of cells without IL-2 subtracted from sample means.

### Assessment of T cell receptor and MHC class II expression

Total RNA was extracted from 14 day T cell and BM-APC cultures (Versagene RNA, Gentra Systems, Minneapolis, MN) and converted into cDNA. Class II expression of the bone marrow cells was assessed by PCR using a forward primer from exon 2 and a reverse primer that overlaps the boundaries of exons 2 and 3 of deer mouse I-Eβ (Table 1[50]). The amplified fragment was cloned and sequenced as described above. T cells were defined by PCR amplification of the TCRβC chain using the primers listed above (forward, exon 1; reverse, exon 2). β-Actin expression was assessed for each population.

### ELISA serology

Sera were collected at euthanasia by cardiac puncture. Plates were coated with 5 μg/ml KLH in PBS overnight and washed 5× with wash buffer (PBS-0.1% TWEEN-20). Plates were then blocked with blocking buffer (5% nonfat powdered milk in wash buffer) for 1 hour at room temperature. The sera and remaining reagents were diluted in blocking buffer. Sera were incubated in duplicate for 2 hours at room temperature, followed by goat anti-*P. leucopus *IgG (H&L) (KPL, Gaithersburg, MD) for 1 hour, then horse anti-goat IgG-HRP conjugate for 1 hour (Vector, Burlingame, CA). ABTS substrate (Sigma) was incubated for 15 min, and plates were read at 414 nm. Means were calculated with the background (1:100 normal deer mouse serum) subtracted.

### In vitro helper T cell expansion

In vitro stimulation of helper T cells was performed essentially as described elsewhere [56,57]. Lymph nodes from immunized deer mice were made into single-cell suspensions by gently disrupting the capsule between the ends of sterile frosted microscope slides. The cells were washed twice in HBSS and plated at 5 × 10^6 ^cells per well (24 well plate) with 20 μg/ml KLH in DMM-5. After a 4 day incubation, the lymph node cells were collected and washed twice in DMM-5. The number of recovered lymph node cells varied between animals, but between 2 × 10^5 ^and 5 × 10^5 ^cells were recovered and plated with fresh antigen and 3 × 10^6 ^thawed autologous splenocytes in DMM-5 in 24 well plates in 1 ml of DMM-5. At 2 day intervals, cultures were fed by removing 750 μl of media and replacing it with DMM-5 containing 20 U/ml of recombinant human IL-2. When cultures were greater than 80% confluent, cells were passaged 1:2 into additional wells or into T25 tissue culture flasks. This process was continued for 14 days to expand T cells. Cultures were also restimulated at two week intervals with fresh mitomycin-C treated autologous splenocytes as above, or 2 × 10^6 ^BM-APC to continue propagation of T cell lines.

### Functional assessment of bone marrow-derived APC

BM-APC were examined for functional capacity to process and present KLH to sensitized autologous deer mouse T cells expanded in culture. For these experiments, mitomycin-C treated BM-APC (10^4^) or splenocytes (2 × 10^5^) were used to stimulate 10^5 ^T cells in the presence of KLH in 96-well plates. In other experiments, the effects of house mouse tumor necrosis factor (20 ng/ml) were evaluated on APC morphology and capacity to stimulate T cell proliferation. Lastly, antisera to KLH were produced in deer mice and used to assess the capacity of the BM-APC to capture and process antigen for presentation to T cells. In these experiments, antiserum or normal deer mouse serum were diluted to 1:2,000, a saturating dilution in ELISA as described above, with KLH and incubated for 1 hour at room temperature. BM-APC and T cells were then added and the cultures incubated for 72 hours prior to determining proliferative responses by MTS. Means and standard deviations were calculated from duplicate samples.

## List of abbreviations

HFRS, hemorrhagic fever with renal syndrome; HCPS, hantavirus cardiopulmonary syndrome; APC, antigen presenting cell; SNV, Sin Nombre virus; GM-CSF, house mouse GM-CSF; DC, dendritic cell; CFA, complete Freund's adjuvant; RT-PCR, reverse transcription polymerase chain reaction; MTS, 3-(4,5-dimethylthiazol-2-yl)-5-(3-carboxymethoxyphenyl)-2-(4-sulfophenyl)-2H-tetrazolium

## Authors' contributions

BD conducted bone marrow cell culture work. DGW and TAC performed RT-PCR experiments. JP cloned and sequenced the TCRβ cDNA. RMF cloned and sequenced the MHC class II cDNA. TS immunized deer mice and generated T cell lines.
